# Infantile scurvy as a consequence of agricultural intensification in the 1st millennium BCE Etruria Campana

**DOI:** 10.1038/s41598-023-48455-0

**Published:** 2023-12-04

**Authors:** Rachele Simonit, Ségolène Maudet, Valentina Giuffra, Giulia Riccomi

**Affiliations:** 1 Division of Paleopathology, Department of Translational Research and New Technologies in Medicine and Surgery, University of Pisa, Pisa, Italy; 2 History Department, Université du Mans, Le Mans, France

**Keywords:** Anthropology, Archaeology, Metabolic disorders

## Abstract

The 1st millennium BCE in Italy was a time of agricultural intensification of staple cereal production which shaped sociocultural, political, and economic spheres of pre-Roman groups. The lifeways and foodways of the Etruscans, the greatest civilization in western Europe before Roman hegemony, are traditionally inferred from secondary written sources, funerary archaeology, archaeobotany, and zooarchaeology. However, no direct data extrapolated from the study of human skeletal remains are available to evaluate the extent to which agricultural intensification and decreased dietary diversity impacted health and the expression of skeletal indicators of metabolic disease. Macroscopic and radiological analyses were conducted on an archaeological skeletal sample of non-adults (n = 29) recovered from Pontecagnano (southern Italy) dating to the Orientalizing period (730–580 BCE). This allowed us to identify five cases of scorbutic non-adults and to assign diagnostic values to skeletal lesions of scurvy that have not been previously described in the literature. The onset of scurvy in the examined sample is related to the increased reliance of Etruscans on crops lacking vitamin C in this period of agricultural intensification. The skeletal expression of scurvy varied among the non-adults, with differences in location and disease severity; these were interpreted considering the age-at-death of the individuals coupled with feeding behaviors and interindividual variability.

## Introduction

Prior to the political and economic unification of the Mediterranean region by the Romans in the Classical period, Italy was a patchwork of distinct ethnic communities, each with its own language and sociocultural identity. Among these, the Etruscans are considered to have been one of the greatest civilizations in western Europe and are recognized as the precursors of the western Roman Empire^1^. The Etruscans dominated different parts of north-central Italy and the Campania region (southern Italy) where they dynamically exerted their economic, political, and cultural influence within and beyond the Italian peninsula between the 8th and the third centuries BCE. Despite the key role of Etruscan culture in setting the stage for subsequent pan-European trading networks and Roman power in Italy, remarkably little is known about their life conditions. To date, knowledge about Etruscan life, socio-economic and political aspects mainly derive from excavations of cities, sanctuaries, necropolises, and the analysis of material culture^2^. Archaeobotany^3^ and zooarchaeology^4^ have only recently begun to provide information on consumable plants contributing to Etruscan diet and animal husbandry strategies. It is inferred from secondary sources that Etruscan diets relied on domesticated cereals and had poor dietary diversity^5^. This limited dietary range could have contributed to deficiencies in numerous essential micronutrients with deterioration of health conditions, a trend widely seen with the adoption or intensification of agriculture among past communities^6^. Nutritional deficiencies can arise from narrow food selection, famine, or overdependence on nutritionally inadequate staple foods^7^. Diets dominated by cereals in particular can lead to micronutrient deficiencies, for example, in iron, zinc, calcium, and vitamins A, B, and C, with corresponding clinical conditions^8–10^. Of these, avitaminosis C or scurvy is one of the best documented metabolic diseases in osteoarcheological assemblages as testified by the growing body of paleopathological research for an overview^11,12^. However, there is currently no research on specific nutritional diseases in ancient Mediterranean Italy. Here, we present the first systematic population-level investigation of infantile scurvy in non-adult sample from ancient Etruria during the 1st millennium BCE, a time of agricultural innovation and intensification of cereal production. It is largely unknown to what extent resource availability and subsistence strategy impacted the health of this pre-Roman group.

**Figure 1:**
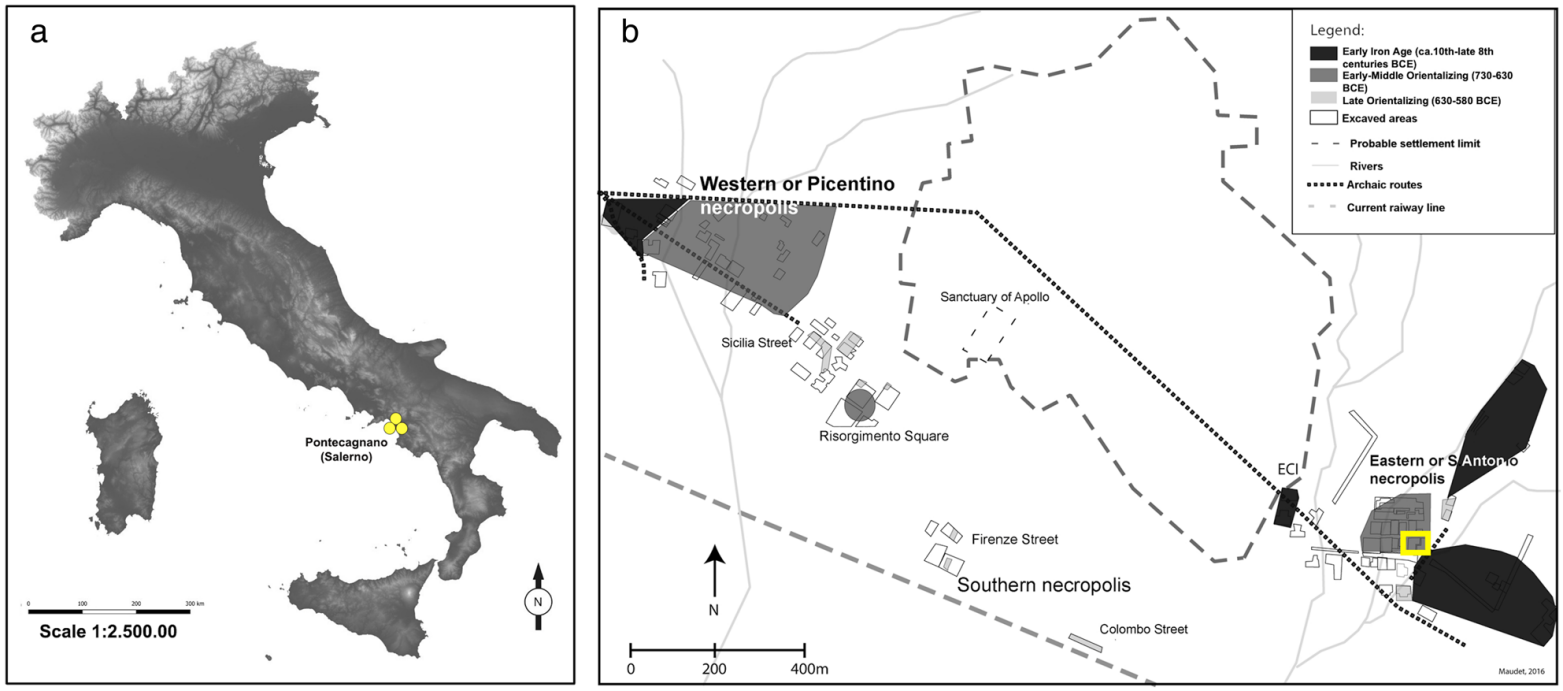
(**a**) DTM (Digital territorial model) of the Italian peninsula, with a marker of the pre-Roman archaeological site of Pontecagnano (yellow dots) located in Campania region (southern Italy) (wms files available at http://wms.pcn.minambiente.it; CC-BY-SA 3.0 license); (**b**) spatial distribution of the site with outlines of the settlement (dashed line), funerary areas (in grey), and location of Chiancone II funerary sector within the eastern or Sant’Antonio necropolis (yellow square) (Courtesy S. Maudet).

The non-adult archaeological assemblage (n = 29) comes from the funerary sector of Chiancone II at the pre-Roman site of Pontecagnano (Campania, southern Italy) (Fig. 1). The analysis of grave goods (e.g., vessels and metal ornaments) allowed archaeologists to precisely date the use of this sector to the Orientalizing period (730–580 BCE)^13,14^. This period was characterized by the emergence of elite groups who controlled large territories where city-like centers started to appear. They likely amassed wealth through land ownership and agricultural production^15^. From a sociocultural perspective, a reorganization of social groups in funerary practice could be observed. This shift included the incorporation of perinatal and young children in the funerary areas as self-representations of families and the celebration of the parental group, which now assumes a gentilitial character^16,17^. This type of phenomenon is often linked to an ideological shift and the integration of new ideas. The presence of Greeks near Pontecagnano from the middle of the eighth century BCE onwards may have played a key-role in this social shift. From economic and sociocultural perspectives, the cohort from Chiancone II sector is an ideal sample for investigating the extent to which pre-Roman agricultural communities were reliant on domesticated crops and the resulting health consequences, especially for infants and children who are at increased risk of micronutrient deficiency.

### Pathophysiology and skeletal manifestation of scurvy

Avitaminosis C is caused by severe vitamin C (L-ascorbic acid or ascorbate) deficiency in the diet or impaired intestinal absorption. Food sources rich in vitamin C include citrus fruits and vegetables (e.g., tomatoes, cabbage, lentils, lettuce). Meanwhile, a diet rich in carbohydrates can impair the absorption of vitamin C as sugars and ascorbic acid vie with each other for assimilation in the intestine^18^. The recommended dietary allowance (RDA) of ascorbic acid is 90 mg/day for men and 75 mg/day for women. This provides ascorbate reserves for one to three months. Pregnant and lactating females have increased requirements of 80–85 mg/day and 115–120/day mg, respectively. Children vary considerably in their daily requirement of vitamin C. The RDA for infants aged 0–12 months is between 40 and 50 mg/day, for children aged 1–3 years 15 mg/day and for children aged 4–8 years it is 25 mg/day^19^ with incremental increases during adolescence according to reproductive maturity. Vitamin C concentrations in human tissues in descending order are the pituitary gland, adrenal glands, eye lens, liver, spleen, brain, pancreas, kidneys, lungs, and skeletal muscles^20^.

The importance of vitamin C in the diet stems from its pivotal role in various bodily functions such as the synthesis of collagen, catecholamines, cortisol, neurotransmitters, antioxidant protection, and amidation of peptide hormones, as well as immune cell functions and maintenance of vascularization^21,22^. The first clinical symptoms of scurvy are limited to lethargy, weakness, fatigue, and irritability^21,23^. When vitamin C has been eliminated from the diet for at least three months, and when body stores fall below 350 mg, all signs associated with scurvy are related to dysfunctions of the vascular endothelium, resulting in fluid extravasation and hemorrhage. Musculoskeletal disorders are attested in 80% of scurvy cases in the form of swollen joints, arthralgia, myalgia, hemarthrosis, hematomas, and subperiosteal hemorrhages associated with the stimulation of new bone formation due to loosely attached periosteum overlying the limbs^21,24^. Other manifestations related to vascular fragility include, for example, ‘vascular purpura’, ecchymosis, and poor wound healing (lack of ascorbic acid reduces platelets)^22–25^. Ophthalmologic problems stemming from hemorrhages include petechiae of the eyelid and proptosis^26–28^. Gingival mucosal changes are also reported in the form of discoloration, petechiae, and gum hypertrophy. Finally, dental problems, like periodontal disease and tooth loss, due to hemorrhagic gingivitis are also documented^29^. With the introduction of a few grams of vitamin C, spontaneous bleeding ceases within a few hours, muscle and bone pain gradually disappear, and gums begin to heal within 2–3 days.

For complete healing, approximately three months of adequate vitamin C intake is required^22,25^. If untreated, avitaminosis C can become chronic and have serious consequences due to oxidative stress, inflammation, organ dysfunction and, in the worst cases, can even lead to death as a result of cardiac, pulmonary, or neurological hemorrhages^22,23,25^.

The typical manifestations of vitamin C deficiency are evident in tissues that contain collagen such as cartilage, osteoid, periosteum, blood vessels and gingival connective tissue. The skeletal manifestations of scurvy are a function of the growth rate of the affected tissues. As a result, bone changes are most often observed in infants and children as they have rapid bone growth compared to adults^24,30^. According to the WHO^31^, pregnant women and children are the most vulnerable to micronutrient deficiency, not only from low dietary intake but, especially, from higher physiological requirements. In the early phases of scurvy, vitamin C deficiency is responsible for defective osteoid matrix formation. This results in increased trabecular and cortical resorption at the junctions between cartilage and diaphyses in the bones of infants and children^30^. These changes have a symmetrical distribution and involve multiple bones, in particular the femora and tibiae.

Such skeletal manifestations of active scurvy are recognizable with radiological analysis. A suite of distinct signs can be seen on the long bones in the form of (1) a wide and thick sclerotic metaphyseal line due to calcified cartilage (or white line of Fränkel); (2) a hypodense transverse area above the white line of Fränkel due to trabecular rarefaction, known as ‘scurvy line’ (or Trümmerfeld zone); (3) microfractures involving the lateral aspects of the white line of Fränkel with resulting triangular defects (corner sign or corner sign of Park); (4) cortical thinning (i.e., ‘pencil-point’ cortex); and (5) metaphyseal widening and osteopenia with a ground glass appearance^21,24,32,33^. In contrast, the healing stage of the disease is recognizable with x-rays in the form of a small beak-like excrescence, a result of repaired microfractures on the metaphyseal plates (i.e., Pelkan’s spur), and an increased density around the epiphysis (i.e., Wimberger ring)^34^.

Collagen impairment that affects the endothelium of small blood vessels cause capillary fragility and chronic bleeding. In regions where blood vessels are superficial or in areas where muscle contractions can damage the walls of already weakened blood vessels, the osteological response to bleeding provides the most important means of detecting scurvy in paleopathology^11,12,35^. The result of this process is an inflammatory response with increased capillary formation which causes abnormal vascular impressions (ABVIs), and periosteal or subperiosteal new bone formation (SPNBF) accompanied by localized fine porosities in bone tissue. These porosities, typically < 1 mm in diameter, penetrate the compact bone and are visible with or without low magnification^35,36^. The alterations are commonly found on the areas of bones where there are anatomical connections between bones, vasculature, and muscular actions. For example, scorbutic lesions can be found on the greater wings of the sphenoid bone, parietal bones, zygomatic bones, maxillae, and mandible at the insertion and origin points of the muscles used for mastication. Post-cranial bones such as the scapulae and the ilia are also sites of scorbutic lesions due to the underlying bleeding that can occur at the insertion and origin areas of muscles used for adduction and abduction of the shoulder and hip joint respectively^12,35–41^.

Although subperiosteal hemorrhages and hematomas occur during the active stage of scurvy, SPNBF remains well visible on X-rays even after vitamin C is re-introduced into the diet (i.e., convalescence and recovery). The differential expression of scorbutic skeletal lesions can be used as a proxy to infer the progress of the disease between active and healing phases^34^.

## Results

### Osteological analysis

The examination of completeness using the API and QBI revealed that there was a sufficient quantity of osseous material for almost the entire non-adult cohort, with 62.1% (18/29) of the non-adult sample categorized as API class 2 and 24.1% (7/29) as API class 3.

API class 4 and 5 are considered well-preserved bones. Only one non-adult (3.4%) was categorized as class 4 (i.e., 50–74% preserved) and three non-adults (10.3%) as class 5 (i.e., 75–99% preserved). Sufficient cortical preservation represented by QBI class 2 and 3 were present in 55.2% (16/29) and 34.5% (10/29) of the sample, respectively.

Age-at-death was estimated for the entire non-adult cohort. Age distribution revealed that more than half of the individuals were in early childhood (58.6%, 17/29) and the second largest age group were infants (24.1%, 7/29*)*, followed by four late children (13.8%) and one fetus (3.4%).

### Paleopathological analysis

**Figure 2:**
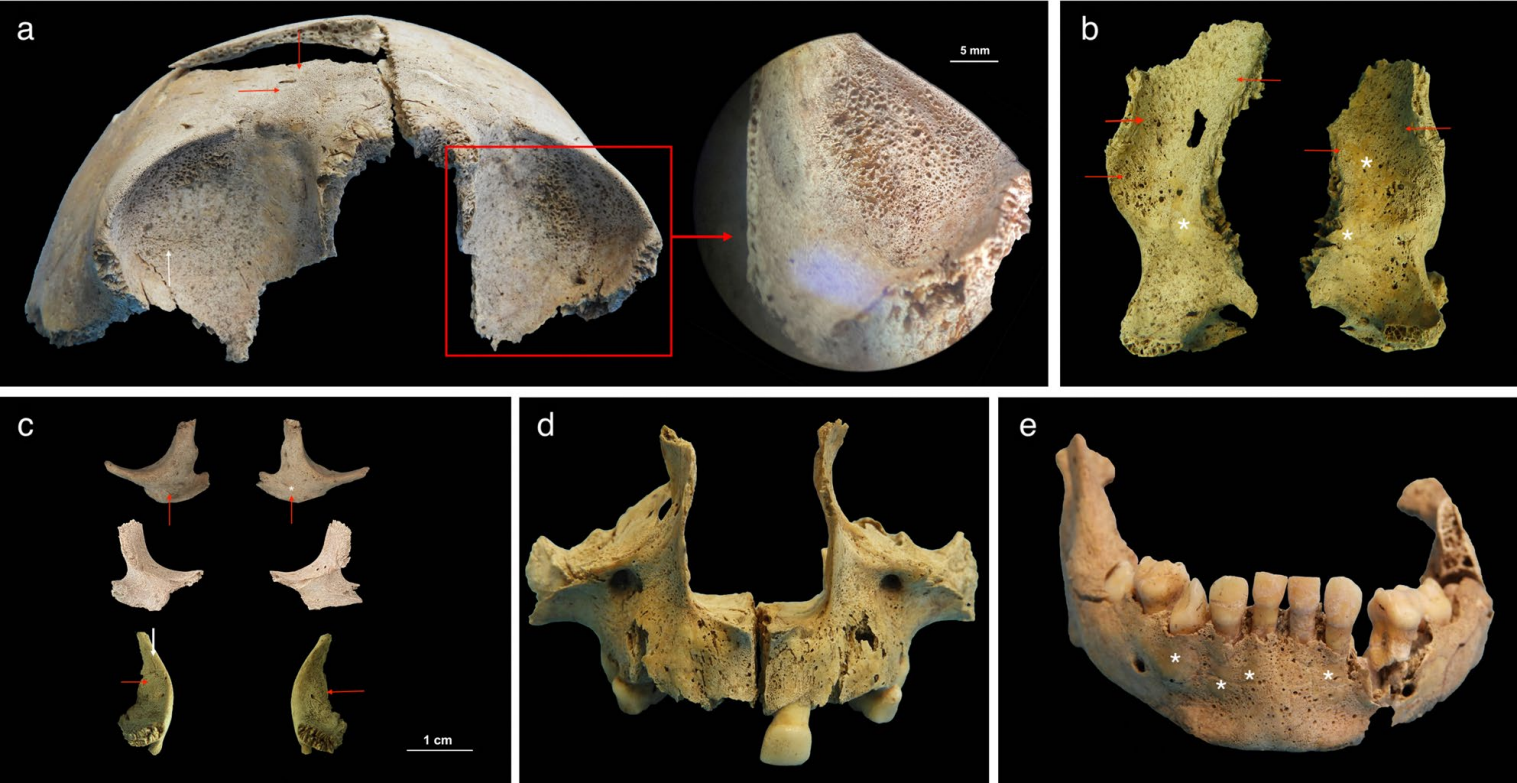
PC4475 (1.5–2.5 years). (**a**) Anterior view of the frontal bone with bilateral abnormal porosities and asymmetrical discrete areas of new bone formation (with no accompanying thickening of the diploë) on the superior orbits. The grey bone area with associated blood vessel impression (white arrow), prominent in the right orbit, was interpreted as hematoma remnants. New bone layers in the glabellar region were also visible (red arrows). Superior view of the left orbit (red square) shows mass of vascular cortical bone formed as rapid response to inflammation; (**b**) lateral surface of the greater wings of the sphenoid bone with bilateral SPNBF (red arrows) attached on the original cortex (*); (**c**) lateral view (top) and medial (middle) of the zygomatic bones with bilateral and symmetrical abnormal cortical porosities < 1 mm and SPNBF (red arrows) compared to original cortex (*). The orbital surfaces (bottom) showed vascular impressions (white arrow) and SPNBF (red arrows) giving a hypertrophic appearance to the bones; (**d**) anterior view of the maxilla with bilateral, symmetrical and diffuse SPNBF in correspondence of the infraorbital foramina and extending along the lateral borders of the nasal aperture, and maxillary alveolar process; (**e**) anterior view of the mandible with SPNBF involving large areas of the mental eminence and of the alveolar process with erupted teeth; new bone layer was visible compared to the original cortex (*). Photo R.S. and G.R.

**Figure 3:**
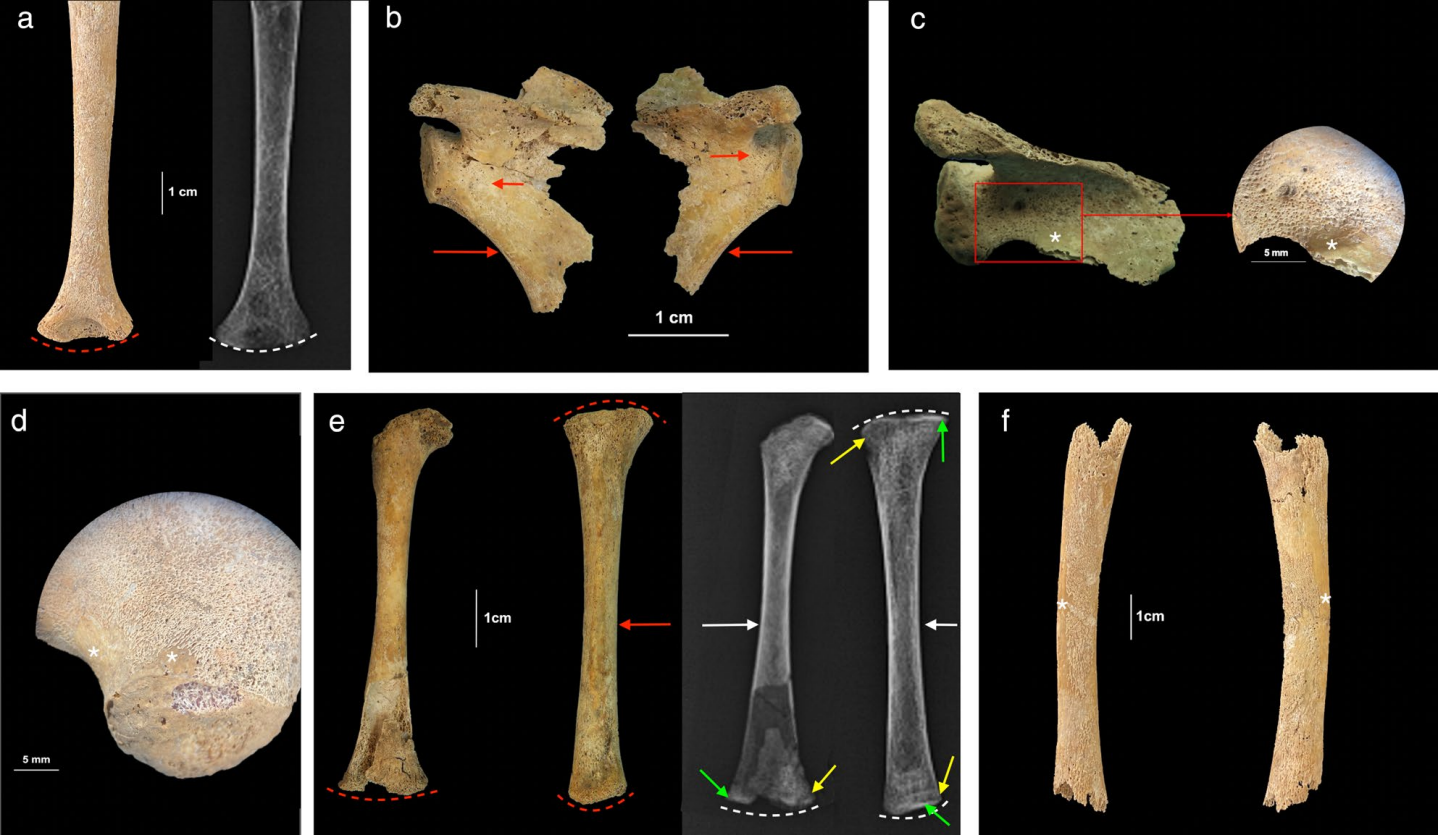
Individual PC4475 (1.5–2.5 years). (**a**) Posterior view of the right humerus with diffuse SPNBF and evidence of mild metaphyseal enlargement (red dashed line), also confirmed by x-ray (white dashed line); (**b**) dorsal view of the scapulae with bilateral abnormal porosities < 1 mm in diameter penetrating cortical bone of the *infraspinatus fossae* and SPNBF, also along both axillary margins (red arrows); (**c**) superior view of the right scapula with SPNBF on the *supraspinatus fossa* and close-up (red square) of the layer of new bone formation compared to the original cortex (*); (**d**) gluteal surface of the right ilium showing diffuse SPNBF attached to the original cortex (*); (**e**) anterior view of the right femur and tibia showing distal metaphyseal enlargement of the former and enlargement of both ends in the latter (red dashed lines). The tibia exhibited diffuse SPNBF on the medial side (red arrow). X-ray confirmed the metaphyseal defects (white dashed lines) and revealed distal Fränkel white lines (green arrows) and ‘scurvy lines’ (or Trümmerfeld zone) in the right femur and in both extremities of the tibia (yellow arrows). The presence of ‘pencil thin cortex’ of the femoral diaphysis (white arrow) was also radiologically visible as well as the presence of pathological SPNBF of the tibia (white arrow). Generalized ground-glass appearance of the long bones was visible with osteopenia in the proximal tibia; (f) Lateral (left side) and medial (right side) surface of the left tibia preserved in its diaphysis showing SPNBF attached to the original cortex (*). Photo R.S. and G.R.

**Figure 4:**
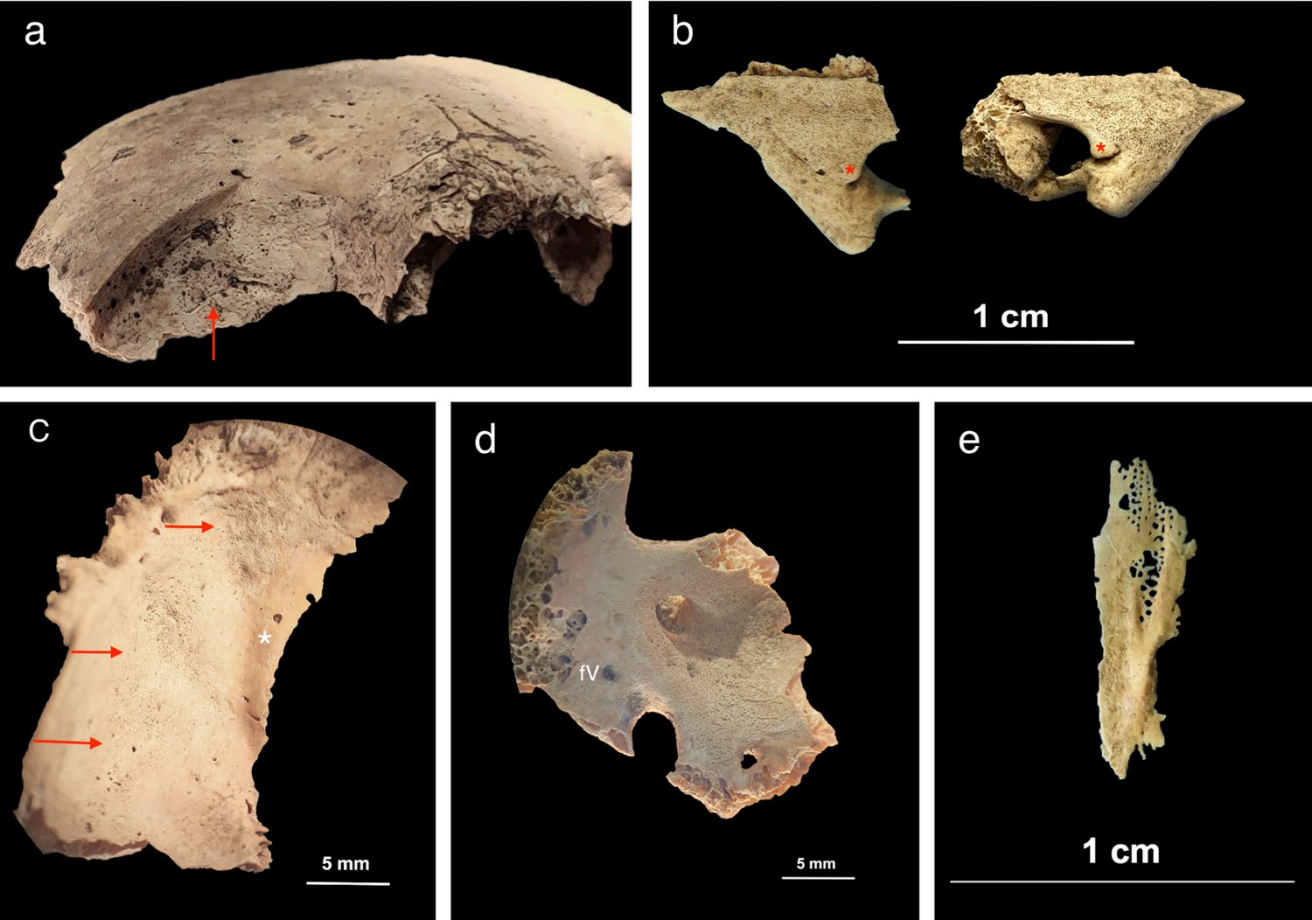
Individual PC4684 (2.5 years). (**a**) Anterior view of the frontal bone with discrete bone outgrowth on the superior part of the right orbit compatible with residual hematoma (red arrow); unfortunately, the left side was not sufficiently preserved to ascertain the bilaterality; (**b**) superior view of the lesser wings of the sphenoid bone with bilateral SPNBF attached to the original cortex (*); (**c**) cerebral surface of the superior part of the right greater wing of the sphenoid bone with evidence of SPNBF (red arrows) attached to the original cortex (*); (**d**) cerebral surface of the inferior part of the right greater wing of the sphenoid bone with SPNBF extending around the *foramen rotundum, ovale* and *spinosum* but sparing the small sphenoidal emissary foramen, or *foramen Vesalii* (fV); (**e**) superior view of the pterygoid plate (side unknown) with abnormal porosities < 1 mm in diameter penetrating cortical bone. Photo R.S. and G.R.

**Figure 5:**
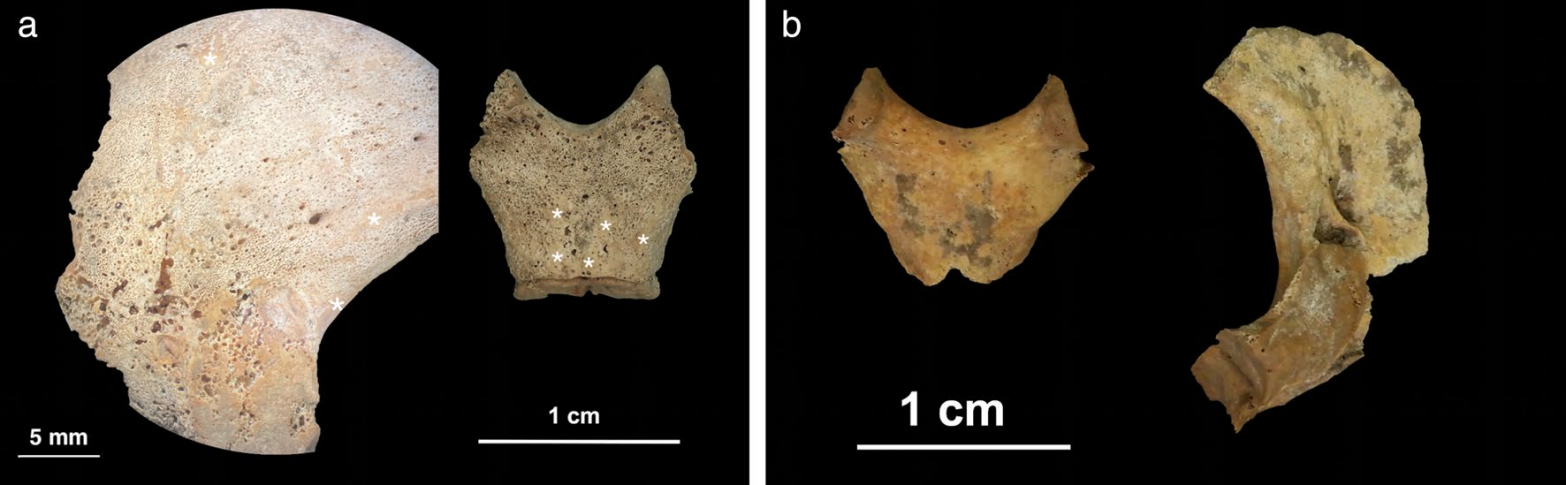
Individual PC4475 (1.5–2.5 years). (**a**) External surface of the right *pars lateralis* with diffuse SPNBF attached to the original cortex (*) and of the inferior surface of the *pars basilaris* with SPNBF attached to the original cortex (*); (**b**) comparison with healthy *pars basilaris* and right *pars lateralis* (external surface) showing no SPNBF in another individual PC4689 with the same age-at-death (1.5–2.5 years). Photo R.S. and G.R.

**Figure 6:**
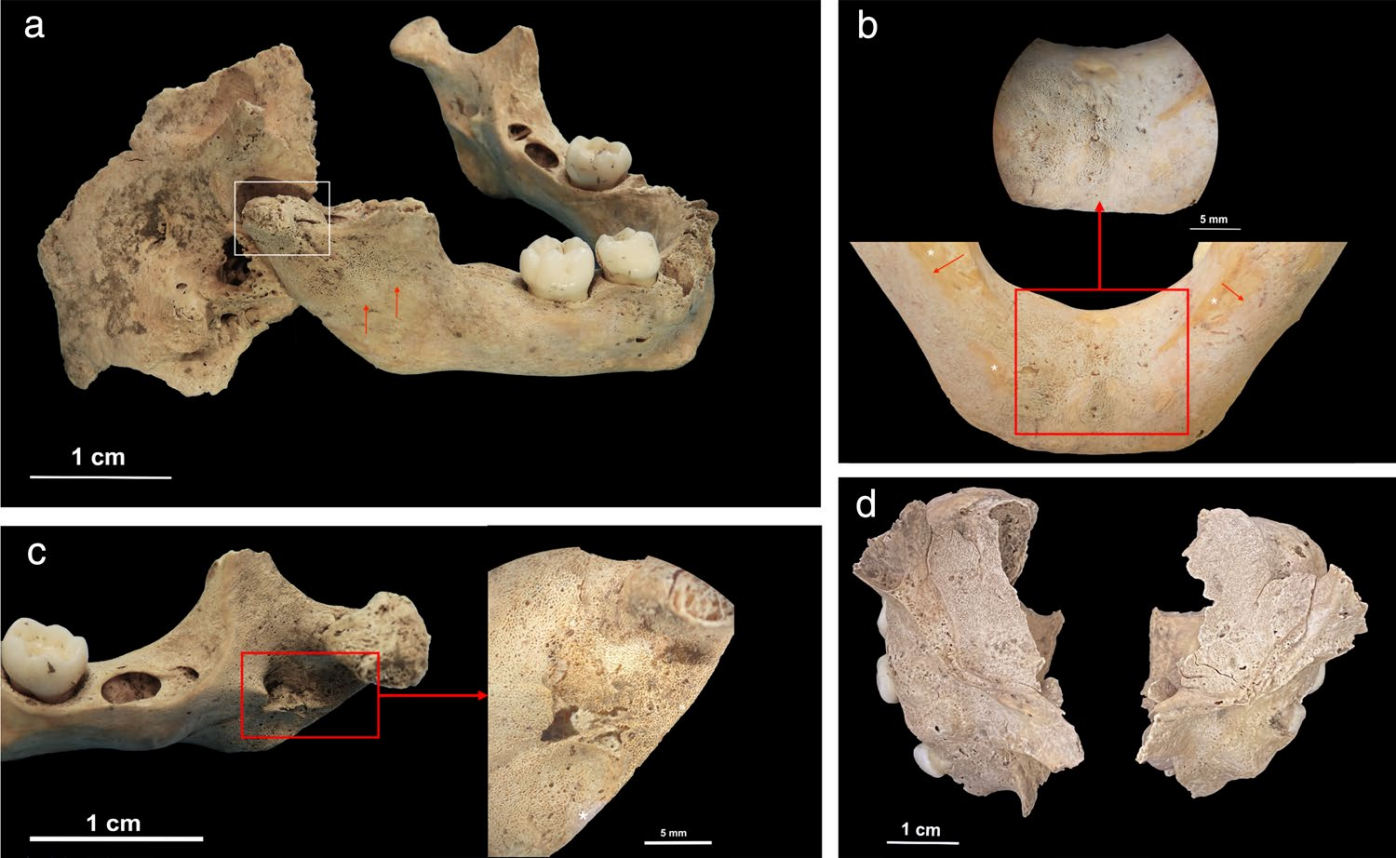
Individual PC4684 (2.5 years). (**a**) Lateral view of the right temporomandibular joint with evidence of fracture involving the neck and head of the right condylar process (white square) and SPNBF of the external surface of the mandibular ramus extending half of its length (red arrows); (**b**) internal side of the mandible with diffuse SPNBF on the inferior and superior mandibular spines (red square) but also exhibiting grey deposits of SPNBF along the inferior borders bilaterally (red arrows) compared to the original cortex (*); (**c**) medial surface of the right ramus of the mandible with diffuse SPNBF extending along the coronoid process, mandibular foramen, mylohyoid groove and gonion region. Close-up of SPNBF and remanent of original cortex (*) (red square); (**d**) superior view of left and right orbital surfaces of the maxilla showing diffuse SPNBF. Photo R.S. and G.R.

Macroscopic lesions in the form of abnormal porosities < 1 mm in diameter that penetrate the cortical bone and/or SPNBF were identified on 5/29 non-adults (17.2%) aged 2–6 years. These individuals exhibited various states of preservation (Table 1). The location, type, and distribution of the lesions documented on the five non-adults are presented in Table 1. The lesions of the skull were located on the sphenoid bone (e.g., greater and lesser wings, foramen *rotundum,* and foramen *ovale*), zygomatic bones (posteromedial, lateral and orbital surfaces), maxilla (posterior and anterior surface, infraorbital foramina, and orbital surface), and mandible (medial surface of the ramus/coronoid process and the superior and inferior mental spines) (Figs. 2, 3, 4, 5 and 6).

**Table 1:** Distribution of macroscopic and radiological lesions in the five non-adults from the Chiancone II funerary sector.

	Lesion location	PC4475	PC4541	PC4633	PC4684	PC4689
		1.5–2.5 years	5.5–6.5 years	5.5–6.5 years	2.5 years	1.5–2.5 years
		API/QBI 5/4	API/QBI 2/3	API/QBI 3/3	API/QBI 5/5	API/QBI 3/3
Diagnostic	Sphenoid bone: greater wings (lateral and cerebral surfaces)	X	\	X	X	X
	Sphenoid bone: lesser wings	X	\	0	X	X
	Sphenoid bone: body	\	\	\	\	\
	Sphenoid bone: pterygoid fossae/plates	\	\	0	X	\
	Sphenoid bone: foramen rotundum (cerebral surface of the greater wings)	X	X	0	X	X
	*Sphenoid bone: foramen ovale (cerebral surface of the greater wings)*	X	X	X	X	0
	*Sphenoid bone: foramen spinosum (cerebral surface of the greater wings)*	\	\	0	X	\
	*Occipital bone: external surface of partes laterales and pars basilaris*	X;X	\;0	0; \	0;\	0 (R), \ (L);0
	Temporal bones: lateral surface of the squama	X	0	\	\	X
	Zygomatic bones (posteromedial, orbital and lateral surface)	X	0	X	X	X
	*Zygomatic bones (orbital surface)*	X	\	0	X	X
	Maxilla: anterior surface/infraorbital foramina and posterior surface	X	\	X	X	X
	Maxilla and palatine processes: hardpalate surface	X	\	X	0	\
	Mandible: medial surface of the ramus/coronoid process	X	0	X	X	X
	Mandible mylohyoid line	0	\	0	0	0
	*Mandible*:* inferior and superior mental spines*	X	\	0	X	X
	Scapulae: supraspinous fossa and infraspinatus fossa	X	\	X	X	\
	*Scapulae: axillary margin*	X	\	0	0	\
	Ilium: gluteal and medial surface	X	\	0	X	\
Highly consistent/Ti| typical	Ectocranial surface of cranial vault (frontal bone, parietals and occipital bone)	X	0	0	0	0
	Frontal bone: orbital roof	X	\	\	X (L); \ (R)	X
	Ribs: costochondral joints	\	\	\	\	\
	Long bones: metaphyseal abnormal porosity	0	\	X	0	\
	Long bones diaphysis	0	\	X	X	0
Consistent with	Endocranial surface	X	X	0	X	X
	Ribs shaft	X	\	0	0	0
	Postcranial skeleton: diaphysis of long bones (upper and lower limbs)	X	\	X	X	X
	Metaphyseal enlargement	X	\	X	X	\

	Radiographic Features	PC4475	PC4541	PC4633	PC4684	PC4689
		1.5–2.5 years	5.5–6.5 years	5.5–6.5 years	2.5 years	1.5–2.5 years
	White line of Fränkel	X	\	\	X	\
	Scurvy line (or Trümmerfeld zone)	X	\	\	X	\
	Pelkan spur	0	\	\	0	\
	Corner sign of Park	0	\	\	0	\
	Long bones ground glass osteopenia	X	\	0	X	X
	Pathological SPNBF postcranial skeleton: diaphysis long bones (upper and lower limbs)	X	\	X	X	X
	Metaphyseal enlargement	X	\	X	X	\

Lesions are bilateral unless specified. Macroscopic skeletal features derived from the present study are in italics.

API, anatomical preservation index; QBI, qualitative bone index, X, presence of lesion; 0, absence of lesion; \, not observable; R, right side; L, left side.

SPNBF located on the superior and inferior mental spines of the mandible was observed in three children aged between 1.5 and 2.5 years (PC4475, PC4684, PC4689). Individual PC4684 also exhibited evidence of a fracture involving the head and neck of the right mandibular condylar process. The presence of woven bone indicated that the fracture had begun to heal (Fig. 6).

Individuals PC4475 (1.5–2.5 years) and PC4684 (2.5 years) exhibited diffuse SPNBF on their entire mandible (Fig. 2). The dental and skeletal age-at-death estimations revealed a 1.2–year discrepancy for PC4475 and a 1.0–year discrepancy for both PC4684 and PC4689 between the two methods.

Macroscopically, PC4475 (1.5–2.5 years) also exhibited SPNBF of the inferior surface of the *pars basilaris* and bilateral SPNBF of *partes laterales* on their occipital bone (Fig. 5).

Abnormal bilateral porosities < 1 mm in diameter penetrating cortical bone with asymmetrical new bone formation on the orbital roof were observed in individuals PC4475, PC4684, PC4689 (Figs. 2 and 4). Lesions affecting the cranial vault and squama of the temporal bones were observed in one (PC4475) and two individuals (PC4475 and PC4689), respectively (Table 1).

Macroscopic alterations of the post-cranial skeleton included bilateral and symmetrical abnormal penetrating cortical porosities < 1 mm and SPNBF of the scapulae’s*infraspinatus* and *supraspinatus* fossae, the axillary margins of the scapulae (new feature, see “Methods” section), and bilateral SPNBF on the ilia (Figs. 3 and 6).

Only one child, PC4633, exhibited abnormal penetrating cortical porosities that extended for 19 mm away from the metaphyseal plate (the range of variation for normal metaphyseal porosity extending from the zone of ossification of the growth plates was 7–9 mm in the observable non-adult cohort (14/29) of Chiancone II).

Macroscopically observable SPNBF was present in four individuals (Table 1) and variably distributed on the upper limbs (Fig. 3a) and lower limbs (Fig. 3f). Radiographic examination revealed osteoid matrix defects at the metaphyseal growth plates and a radiopaque zone in the lower limbs of PC4475 (1.5–2.5 years) and PC4684 (2.5 years) (Fig. 3e).

The radiological analysis confirmed the pathological nature of the SPNBF (> 2 mm) and metaphyseal enlargement in four and three individuals, respectively (Table 1) (Fig. 3e). Finally, radiographically ‘pencil thin cortex’ of the lower limbs was visible for individuals PC4475 and PC4684 (Fig. 3e).

### Differential diagnosis

Differential diagnosis was conducted to account for normal physiological bone growth and pathologies that can cause skeletal lesions similar to those of scurvy including childhood acute leukemia, rickets, and iron-deficiency anemia^22,32,42^.

Endochondral growth of the long bones is responsible for osteoclastic activity at the metaphyseal plates which results in porosity that usually does not continue more than 10 mm from the growth plate, otherwise it is considered pathological^37^. Moreover, long bones also undergo physiological periosteal reactions as part of rapid appositional growth, resulting in bilateral and symmetrical diaphyseal SPNBF. The presence of symmetrical SPNBF on the diaphyses of long bones is common for newborn and infants between one to six months old^42^. The location and morphology of SPNBF documented on the five non-adults from Chiancone II funerary sector allowed us to exclude the possibility of a normal physiological response of the periosteum.

When considering alternative pathologies, childhood acute leukemia is usually signaled by the presence of well-defined osteolytic lesions with no marginal inflammation, enlarged vascular foramina, and periosteal reactions that particularly involve the metaphyseal areas^39^. The morphology of the non-adults’lesions and their distribution, which included the sphenoid bone (Table 1), are inconsistent with a diagnosis of this hematologic malignancy.

Co-occurrence of scurvy with rickets in not uncommon^12,43^. However, the possibility of observing a coexistence of pathologies at the skeletal level is related to the stage of development of the diseases and type of alterations, and co-occurrence can be established if both pathologies are expressed in their typical features^44^. In the present sample, the co-occurrence of scurvy and rickets could be ruled out based on the absence of core features of rickets such as symmetrical bowing of the upper and lower limbs, deformities of the ilium, femoral neck (coxa vara), and mandibular ramus, as well as the absence of any expansion of costochondral junctions (i.e., rachitic rosary)^12^. Finally, a lack of ascorbic acid often causes a scurvy-associated anemia as vitamin C assimilation increases nonheme iron absorption^24^. The porosities observed on the orbits are linked to a wide spectrum of etiologies^45^, but pore features and the absence of increased diploe thickness as a result of marrow hyperplasia^39^ allowed us to exclude anemia.

As a result, the morphology of the lesions, their location, and distribution make infantile scurvy the final diagnosis for the five non-adults at Pontecagnano-Chiancone II funerary sector.

## Discussion

### Interpreting infantile scurvy lesions at Pontecagnano

Differential diagnosis and, especially, the application of a methodological framework that did not set a quantitative threshold for the diagnosis of metabolic disease (see “Methods”) allowed us to observe cases of infantile scurvy with, differing expressions of lesions, in five individuals. these cases exhibited heterogeneous state of preservation and estimated ages corresponding to 1.5–2.5 years (PC4475 and PC4689), 2.5 years (PC4684) and 5.5–6.5 years (PC4541 and PC4633) (Table 1).

There are three main reasons for the interpretation of the location of the macroscopic cranial and post-cranial skeletal alterations as indicative of scurvy in the present study: (1) mastication biomechanics; (2) eye movements; (3) interindividual variability in disease expression. Mastication biomechanics may explain the observation of abnormal bilateral and symmetrical porosities < 1 mm in diameter that penetrate the cortical bone and the SPNBF on the lateral surface of the greater wings of the sphenoid bone, where the temporalis muscle attaches. Conversely, the SPNBF around the *foramina ovalia and spinosa* (new indicators, see “Methods” section) seen in these scorbutic individuals could be explained by localized hemorrhaging where the mandibular nerve, a branch of the maxillary nerve, and meningeal arteries pass through as well as by entheses solicitations during deglutition (Table 2). This phenomenon has been previously reported and discussed in cases where SPNBF is present where neurovascular structures pass through the *foramina rotundi* of the sphenoid bone^46^ and of the infraorbital foramina of the maxilla^35–38^.

**Table 2:** Location of macroscopic cranial and postcranial osseous lesions attributed to infantile scurvy described in the primary paleopathological literature^12,35–41,46,47,52^.

Skeletal features				
Diagnostic strength	Lesion location	Lesion type and distribution	Anatomical connection: musculature (new features only)	Anatomical connection: vasculature (new features only)
Diagnostic	Cranial			
	Sphenoid bone: greater wings (lateral and cerebral surfaces)	Bilateral and symmetrical abnormal porosity < 1 mm in diameter penetrating cortical bone and/or SPNBF		
	Sphenoid bone: lesser wings	Bilateral and symmetrical abnormal porosity < 1 mm in diameter penetrating cortical bone and/or SPNBF		
	Sphenoid bone: body	SPNBF		
	Sphenoid bone: pterygoid fossae/plates	Bilateral and symmetrical Abnormal porosity < 1 mm in diameter penetrating cortical bone and/or SPNBF		
	Sphenoid bone: foramen rotundum (cerebral surface of the greater wings)	Bilateral and symmetrical SPNBF		
	*Sphenoid bone: foramen ovale (cerebral surface of the greater wings)*	Bilateral and symmetrical SPNBF	*Temporalis muscle (indirect) with masticatory function; Tensor veli palatini muscle (external) with deglutition function*	*Accessory meningeal artery and sphenoid emissary vein*
	* Sphenoid bone: foramen spinosum (cerebral surface of the greater wings)*	Bilateral and symmetrical SPNBF	*Tensor veli palatini muscle (external and indirect) with deglutition function*	*Middle meningeal artery and vein*
	* Occipital bone: external surface of partes laterales and pars basilaris*	*Bilateral and symmetrical SPNBF (except for pars basilaris)*	*Rectus capitis lateralis and rectus capitis posterior major muscles (partes laterales of the occipital bone); rectus capitis anterior and longus capitis muscles (pars basilaris of the occipital bone)—Postural muscles for flexion, rotation, extension of the head*	*Vertebral artery, occipital artery, ascending cervical artery, inferior thyroid artery*
	Temporal bones: lateral surface of the squama	Bilateral and symmetrical abnormal porosity < 1 mm in diameter penetrating cortical bone and/or SPNBF		
	Zygomatic bones (posteromedial, and lateral surface)	Bilateral and symmetrical abnormal porosity < 1 mm in diameter penetrating cortical bone and /or SPNBF		
	* Zygomatic bones (orbital surface)*	*Bilateral and symmetrical abnormal porosity* < *1 mm in diameter penetrating cortical bone and /or SPNBF*	*Extraocular muscles for internal eye movements*	*Lacrimal artery, ophthalmic artery, and infraorbital artery*
	Maxilla: anterior surface/infraorbital foramina and posterior surface	Bilateral and symmetrical abnormal porosity < 1 mm in diameter penetrating cortical bone and/or SPNBF		
	Maxilla and palatine processes: hard palate surface	Abnormal porosity < 1 mm in diameter penetrating cortical bone extending markedly into the posterior portion of the palate with spiculated bone		
	Mandible: medial surface of the ramus/coronoid process	Bilateral and symmetrical abnormal porosity < 1 mm in diameter penetrating cortical bone and/or SPNBF		
	Mandible mylohyoid line	Bilateral and symmetrical SPNBF		
	* Mandible: superior and inferior mental spines*	*Bilateral and symmetrical SPNBF*	*Genioglossus muscle (superior spine); Geniohyoid muscle (inferior spine) for tong movements and deglutition*	*Lingual artery*
	Post-cranial			
	Scapula: supraspinous fossa and infraspinatus fossa	Bilateral and symmetrical abnormal porosity < 1 mm in diameter penetrating cortical bone and/or SPNBF		
	* Scapula: axillary margins*	*Bilateral and symmetrical abnormal porosity* < *1 mm in diameter penetrating cortical bone and/or SPNBF*	*Teres major and subscapularis muscles (rotator cuff complex)*	*Subscapular artery branches of the axillary artery*
	Ilium: gluteal and medial surface	Bilateral and symmetrical abnormal porosity < 1 mm in diameter penetrating cortical bone and/or SPNBF; vascular impressions		
Highly consistent/typical	Cranial			
	Ectocranial surface of cranial vault (frontal bone, parietals and occipital bone)	Bilateral (except for frontal bone) abnormal porosity < 1 mm in diameter penetrating cortical with asymmetrical localized SPNBF§ (especially in correspondence of the bosses) and ABVI; no thickening of the diploe		
	Frontal bone (orbital roof)	Bilateral abnormal porosity < 1 mm in diameter penetrating cortical bone and asymmetrical localized SPNBF§§ (i.e., residual haematoma); vascular impressions; no thickening of the diploe		
	Post-cranial			
	Ribs: costochondral joints	Flaring		
	Post cranial skeleton: metaphyseal of long bones	Abnormal porosity < 1 mm in diameter penetrating cortical bone extending beyond 10 mm from the plate		
	Long bones: diaphysis	Bilateral and symmetrical ossified haematomas		
Consistent with	Cranial			
	Endocranial surface	*PADM; ABVI*		
	Post-cranial			
	Ribs: shaft	SPNBF		
	Long bones: diaphysis	Bilateral and symmetrical SPNBF		
	Long bones: metaphyseal enlargement			
Radiographic features				
	White line of Fränkel			
	Scurvy line (or Trümmerfeld zone)			
	Pelkan spur			
	Corner sign of Park			
	Long bones groundglass osteopenia			
	Postcranial skeleton: diaphysis long bones (upper and lower limbs)	Bilateral and symmetrical pathological SPNBF (> 2 mm in thickness)		
	Metaphyseal enlargment			

Details on lesion type and distribution are provided. Further macroscopic skeletal features with details about vasculature and musculature connection and derived from the present study are in italics. Proposed diagnostic strength was established according to the modified Istanbul protocol^48^. Radiological findings of the long bones evaluated in clinical practice are also included^21,24,32–34,49,50^.

SPNBF, subperiosteal new bone formation.

ABVI, abnormal blood vessel impressions^120,121^.

PADM, periosteal apposition of the dura madre^121^.

^§^The connective tissue is the pericranium.

^§§^The connective tissue is the periorbita.

For osseous lesions located on the cerebral surface of the greater wings of the sphenoid bone (Table 2), we propose an additional hypothesis to further explain their presence and diagnostic value. The body tissue with the highest accumulation of vitamin C is the pituitary gland^20^, which is also the most highly vascularized mammalian tissue. Its vascular supply is provided not only by the hypophyseal arteries, but also by a dense network of capillaries^58^.

In the human body, blood vessels have three different types of endothelia that display a remarkable heterogeneity in terms of their morphology, molecular components, and functions^59^. These are: (1) continuous endothelia, which form an uninterrupted barrier between the blood and tissues, are present in the large vessels of the heart and capillaries of the lungs, skin, skeletal muscles, and brain; (2) fenestrated endothelia, with intracellular pores covered by diaphragms, are present in the capillaries of endocrine glands, intestinal mucosa, and kidney peritubular capillaries; and (3) discontinuous or sinusoidal endothelia that have fenestrae, but of larger diameter (i.e., ‘gaps’) than the endocrine vessel fenestrae, and lacking diaphragms; sinusoids are present in the capillaries of liver, kidney glomeruli, and bone marrow^59^.

The intrinsic permeability of fenestrated capillaries enables the rapid exchange of low-molecular weight hydrophilic molecules, peptides, and hormones through the bloodstream^60^. Physiological as well as pathological processes like acute and chronic inflammation or trauma are responsible for increased capillary permeability^60^. Considering the pathophysiology of scurvy, it is attractive to propose the possibility that fenestrated capillaries, near the pituitary gland, affected by impaired collagen synthesis due to vitamin C deficiency, might be prone to increased fragility and thus might more easily become hemorrhagic compared to other tissues or regions of the body.

Scorbutic lesions on the lesser wings of the sphenoid bone were present on some individuals (PC4475, PC4684, PC4689) as well as on the pterygoid fossae/plates for individual PC4684. Repetitive use of musculature implicated in mastication and deglutition (e.g., lateral and medial pterygoid muscles) also explains the lesions seen on the medial surface of the zygomatic bone (where the masseter and temporalis muscles attach indirectly) as well as the lesions observed on various parts of the maxilla and mandible related to the temporalis, genioglossus and geniohyoid muscles (the latter in connection to the mental spines as a new proposed feature). Furthermore, considering the different age-at-death categories represented among the scorbutic individuals, we presume that different feeding behaviors (i.e., the passage from exclusively breastfeeding to the introduction of supplementary solid foods during weaning) had different impacts on the relevant musculature. Masticatory forces from suckling, drinking, chewing, and deglutition are not the same and this shift is expected to be reflected in the absence or differential severity of lesions on the mandible among individuals PC4475, PC4684 and PC4689 aged between 1.5 and 2.5 years. Indeed, between 12 and 18 months, infants become able to coordinate their tongue and jaws for eating and expressing themselves^51^. With this in mind, the more extensive porosities observed on the maxilla and mandible of individuals PC4475 (1.5–2.5 years) and PC4684 (2.5 years) may be indicative of the severity of bleeding at both sites. This may indicate that blood vessels entering the foramina, as well as other soft tissues around these areas, suffered from collagen dysfunctions during the vitamin C deficiency, leading to a substantial vascular response to hemorrhagic processes^52^. Additionally, a healing fracture of the mandibular condyle in individual PC4684 would have negatively impacted the already precarious nutrition of this child as fractures to the mandible result in complications including pain and masticatory dysfunction^53^. A discrepancy (≥ 1 year) was found for the three above-mentioned individuals between dental and skeletal age. Bone development is notoriously susceptible to many factors such as precarious nutritional status, poor sanitary conditions, and diseases that may accelerate or retard bone development^54,55^. This may explain the discrepancy between the two age estimation methods.

Eye movements can explain the pathological signs of the orbital cavity, in particular alterations of the orbital roof of the frontal bone and the orbital surface of the zygomatic bones. The periorbita serves as an attachment site for the extraocular muscles, tendons, and ligaments and it is a support structure for the blood supply to the orbital bones^56^. In contrast to adults, the periorbita in non-adults is loosely attached, making it more prone to tearing and hemorrhaging^57^. Therefore, patients may variably experience orbital and intra subperiosteal hemorrhages that result in the presence of bilateral and symmetrical SPNBF on the orbital surface of the zygomatic bones, as well as asymmetrical bone growth on the superior area of the orbits, which are interpreted as residual hematomas in osteoarchaeological remains.

According to Stark^47^, hemorrhagic reactions from scurvy can exhibit intra-skeletal diversity in terms of location and severity due to variable osteological responses to traumatic events. The latter, along with different ages at onset of symptoms, or the complex interplay between recurrent periods of deficiency, may explain the interindividual variability in scurvy expression among the non-adults under study. This may explain why only one child (PC4475, 1.5–2.5 years) exhibited the new diagnostic cranial lesions consisting of bilateral SPNBF on the *partes laterales* and on the *pars basilaris* of the occipital bone. Similar processes also account for the bilateral involvement of the axillary margins of the scapulae as another new diagnostic feature that was observed in the same individual.

However, a relationship with postural-motor maturational milestones that occur during the first years of life, including the formation of cervical lordosis, and the observed lesions of the occipital bone—where antigravity muscles (Table 2) are solicited to maintain the head in an upright position^61^—might represent a future hypothesis to test.

Finally, we can examine the progression of scorbutic lesions among this sample of non-adults. The five affected individuals who exhibited active lesions at the time of their death in the form of abnormal penetrating cortical porosities < 1 mm on different areas of their skeleton due to chronic hemorrhages also exhibited SPNBF. This indicates the re-introduction of vitamin C into their diet. The diaphyseal hematomas in individuals PC4684 (2.5 years) and PC4633 (5.5–6.5 years) alongside the metaphyseal defects observable radiographically also indicate active stages of scurvy at the time of their death.

### Agricultural intensification during the 1st millennium BCE: the main reason behind scurvy in ancient Etruria

The adoption or intensification of agriculture is well known to have provoked major detrimental effects on health in many different past societies^6,62–67^. A decrease in dietary variability and onset of infantile scurvy has been documented in connection to agricultural transitions during the Neolithic colonization of the Pacific islands^68^, the Early Formative period in northern Chile^69^, and Pre-Neolithic to Neolithic northern Vietnam^70^.

During the 1st millennium BCE in Etruria, signs of such agricultural intensification is attested by archaeological evidence of vegetational changes in pollen assemblages^72^, the emergence of hierarchies of settlement type^71^, different livestock grazing practices^73^, and hydraulic works^74^ that would have supported the primary economic and productive activities, i.e., agriculture and livestock breeding^75^. The recovery of iron tools such as plows, hoes, and spades suggest that these populations had the means to prepare hard soils for planting and to irrigate fields. Farming tools included large sickles, supporting the notion that the main base of agriculture was the cultivation of different cereals. The other pruning tools and pennate axes that were found would have been used for viticulture and arboriculture^76,77^. The intensification of agriculture in Etruria during the 1st millennium BCE should be interpreted within the context of socioeconomic changes. These changes included rapid population growth and the emergence of a network of trades that extended across the whole Mediterranean. Notably, during the Etruscan Orientalizing period (730–580 BCE), competitive aristocrats with ‘princely status’ emerged. Their preeminent status is recognizable through the funerary evidence (i.e., tumuli and rich grave goods)^78,79^. The emergence of hierarchical, centralized societies taking control of long-distance trade and of agricultural labor potentially generated social and food inequalities^80^, thus creating the conditions for possible periodic food shortages and, more significantly, a decrease in dietary variety. This could have ultimately led to deficiencies of key nutrients. Archaeobotanical findings confirm that the Etruscan diet included free-threshed wheat and hulled wheats (i.e., emmer, spelt, einkorn), barley, oat, and rye. These were eaten in the form of flatbreads, soups, and puls/*pulmentum*, a kind of porridge similar to present-day Italian polenta^5^.

Stable isotope analysis of bone collagen of adults and non-adults from the Chiancone II funerary sector (Riccomi et al. 2023, in prep.) and other areas within the site of Pontecagnano^81^, revealed a reliance on C_3_ plant foods. While different types of cereals were consumed, they are all rich in vitamins A, B, and E and lacking in vitamin C^82^.

Of equal importance is the cooking, preparation, and storage of alimentary sources which can impact the vitamin C content of the foods. Exposure to light, oxygen, acidic pH levels, and heat destroys water soluble vitamins like vitamin C. Similarly, drying as a traditional post-harvest process aimed to ensure long-term storage of alimentary sources can also cause deterioration of ascorbic acid^84^.

It cannot be ruled out that fungal infections of plants might have caused crop failure leading to famine or episodes of food crisis resulting in dietary deficiencies^83^.

The natural environment of Pontecagnano may have also contributed to fluctuations in food availability. The territory of the Sele plain, and specifically of the Piana Picentina, despite being very fertile, is an alluvial area particularly prone to land degradation and the formation of swamps. Until the twentieth century, the area was characterized by a humid environment rich in lagoons and lake-palustrine basins^85,86^.

This landscape, dominated by unstable conditions, undoubtedly influenced the quality and quantity of food available to the population that inhabited this area during the Etruscan Orientalizing period. It was only during the Archaic age (end of the 6th and beginning of the fifth century BCE) that impressive hydraulic works were undertaken at Pontecagnano in order to increase cultivable areas^85,87,88^.

The effects of agricultural intensification at Pontecagnano are thus evident when considering the health conditions of these five non-adults. Children’s health is a complex phenomenon that combines biological, behavioral, and environmental forces, and therefore reveals much about a given society^89^.

During their first years of life, the non-adults’ diet and health would have been closely linked to the stages of exclusive breastfeeding and weaning. The positive health impacts of human breast milk attributed to bioactive components (e.g., antibodies, enzymes, proteins and peptides) has been well documented. It is known to offer protection against short and long-term ear, respiratory, and gastrointestinal infections, allergies, and asthma in infants^90^. However, in some cases, breastfeeding could be undertaken by wetnurses or through the administration of animal milk with baby bottles, either due to cultural practices or unavailability of milk from the biological mother. The exclusive use of animal milk as a substitute for breastfeeding can result in vitamin C deficiency in the diet as cow or goat’s milk are deficient in this vitamin^91^.

At the same time, the intake of supplementary foods or liquids other than breast milk during weaning represents a critical phase for the survival of non-adults. An inappropriate quality and quantity of weaning foods, their mixture, and preparation under unhygienic conditions are major factors in the cause of diarrheal diseases in children^92^. At Pontecagnano, supplementary solid foods used for weaning would have been formulated using the available cereals and pulses attested at Etruscan sites. These mixtures were traditionally administered in the form of bread-like or porridge-like foodstuffs^93^. Although weaning foods based on cereals are an excellent source of energy, they are low in ascorbic acid^82^, which could explain the presence of infantile scurvy at Pontecagnano. Additionally, it is well known that vitamin C assimilation is impaired by carbohydrates like glucose, fructose, and sucrose as sugars compete with vitamin C for absorption in the intestine. This fits nicely with past weaning practices for which starch-based weaning foods were often softened using hydromel (i.e., honey and water) or honey^93^.

The high mortality rates during infancy and early childhood seen within the Chiancone II funerary sector (see “Results”) is a proxy not only to infer fertility dynamics, but also to understand maternal health, pathogen load, wider subsistence strategies, and sociocultural aspects of child feeding practices^94,95^. For three non-adults, PC4475 and PC4689 (both 1.5–2.5 years) and PC4684 (2.5 years), it is possible to argue that sociocultural determinants of breastfeeding and past weaning trajectories influenced the intake and malabsorption of vitamin C thus resulting in the onset of scurvy.

A broad comparison of our results with others available in the literature (comparisons did not belong to the same period as the current sample under study), revealed the multifactorial etiology of infantile scurvy. Malnutrition and/or undernutrition linked to breastfeeding and weaning practices have been taken into consideration when discussing infantile scurvy in Bronze Age Britain^96^, Romano-British groups^97^, Byzantine Greece^98^, and late medieval Hungary^99^. Seasonal‐related food scarcity or reduced dietary diversity were likely responsible for avitaminosis C in the early medieval Castel Tirolo (Italy)^100^. Dietary trends, along with food storage and preparation techniques, have also been considered as contributing factors to infantile scurvy in predynastic Egypt^101^, medieval UK^102^, and medieval and post-medieval Poland^103^.

Beyond childhood feeding practices, other factors like environmental stress, socioeconomic structures, food allocation, and food insecurity can be related to the onset of scurvy^104–106^. Environmental instability and resource insufficiency might be the reason for the possible case of infantile scurvy seen in commingled remains from prehistoric Malta^107^. Social control of food distribution was the main reason of infantile scurvy in Romano-British rural contexts^108^ and in the ancient American Southwest^109^. Similarly, dietary restrictions due to a combination of economic constraints and religious prescriptions were crucial contributing factors to the development of scurvy among commingled non-adults from nineteenth century New York^110^. These examples demonstrate how social, economic, and ecological determinants, or a synergy of all three, influence the development of scurvy in the past. Scurvy thus reflects the ecological, economic, cultural, and socio-behavioral forces that shape the diet and nutritional intake of a given society.

For the other two scorbutic individuals, (PC4541 and PC4633) aged 5.5–6.5 years, we assume weaning was completed. Therefore, evidence of scurvy can be interpreted as stemming from dietary monotony where staple crops lacked essential vitamins and there was a limited contribution of other food sources to the overall diet.

Although the cultural influence on infant and young child feeding likely represents the most common cause of infantile scurvy in the past^11^, there are other factors responsible for susceptibility to scurvy, including genetic predisposition, nutrient malabsorption, environmental pollutants, and pathogen load^111–113^.

According to clinical literature, scurvy may arise from other conditions, such as malaria^114,115^. The marshy lands at Pontecagnano in the period under study, present a plausible environmental predisposing factor for the onset of scurvy.

Environmental factors may have thus played a role in the development of metabolic diseases in the non-adult cohort from Chiancone II beyond nutrition, especially for the older children.

## Methods

### Osteological analysis

The osteological analysis was conducted on the non-adult cohort of Chiancone II (n = 29). The state of completeness and preservation of the remains was quantified according to the Anatomical Preservation Index (API) and the Qualitative Bone Index (QBI)^116^. Age-at-death was estimated according to dental eruption and development^117^. Skeletal age was also estimated according to diaphyseal measurements and epiphyseal stage union^118^. The mean age of each individual was assigned to a single age category^118^ for demographic representation: fetuses; 0–1 year (infant), 2–6 years (early childhood); 7–12 years (late childhood). Because the non-adults belonged to pre-pubertal stages of development, sex assessment was not performed.

### Paleopathological analysis

The identification of infantile scurvy in the present study was based on the comprehensive list of features reported by Snoddy et al.^40^ with some modifications, which are described below and reported in Table 2.

- First, we attempt to improve the approach to macroscopic diagnosis of infantile scurvy by clarifying some terminological, conceptual, and methodological aspects as follows: (1) to better specify the morphology of orbital and ectocranial cranial lesions related to scurvy according to Klaus^39,119^; (2) to apply the term ‘periosteal apposition of *dura madre* (PADM)’ for proliferative endocranial lesions instead of the term ‘periosteal apposition’, which is commonly used for long bones and ectocranial surfaces, as there is no proper periosteum covering the endocranial surface. PADM refers to endocranial lesions characterized by the apposition of newly formed bone with a fibrous and porous appearance^121^; (3) to apply the term ‘abnormal blood vessel impressions (ABVI) for short, meandering branches on the endocranium and ectocranial surface of cranial vault related to inflammatory and/or hemorrhagic processes as a result of secondary angiogenesis^120,121^; (4) to exclude porosities on the inferior surface of *pars basilaris* as a potential feature connected to scurvy as it could be related to physiological endochondral growth^122,123^; (5) to consider subperiosteal new bone formation (SPNBF) of the long bones as ‘consistent with’ rather than ’diagnostic of’ scurvy given its association with a wide range of conditions ranging from physiological responses due to rapid bone growth, to infectious and inflammatory diseases, congenital disorders, and metabolic conditions (e.g., scurvy, rickets, hypervitaminosis A)^50,124^. In light of this, SPNBF of long bones was radiologically evaluated in accordance with clinical literature to establish whether SPNBF was physiological (< 2 mm) or pathological (> 2 mm)^49,124^.
- Second, we employed the biological approach^35–38,125^ to test the hypothesis that other bone regions would also be affected by the formation of macroscopically observable osseous scorbutic lesions in the non-adult cohort as a result of anatomical connections between vascular networks and muscular function. These are: (1) SPNBF of the inferior surface of the *pars basilaris* and *partes laterales* (bilaterally) of the occipital bone which is associated with the unique functional complex related to head postural muscles (i.e., *rectus capitis lateralis, rectus capitis posterior major*, *rectus capitis anterior, longus capitis* muscles); (2) bilateral and symmetrical SPNBF around the *foramen ovale* and *spinosum* of the greater wings (cerebral surface) of the sphenoid bone; (3) bilateral and symmetrical SPNBF of the orbital surface of the zygomatic bones in response to stimulation of the periorbita, the orbital periosteum covering all seven *bones* of the orbital cavity; (4) SPNBF of the superior and inferior mental spines (genial tubercles) of the mandible due to geniohyoid and genioglossus muscles solicitated during deglutition and tongue movements; (5) bilateral and symmetrical SPNBF along the axillary margins of the scapulae related to *teres major* and *subscapularis* entheses as part of the rotator cuff muscular complex.
- Third, diagnostic certainty of macroscopic features related to infantile scurvy was established following a careful differential diagnosis for each of the recorded lesions. This was especially so for lesions that are common in a wide range of other conditions such physiological growth, pellagra, rickets, and iron-deficiency anemia. Diagnostic strength was thus defined using the modified Istanbul protocol^48^ as follows: diagnostic, highly consistent/typical, and consistent with.
- Fourth, no quantification and threshold diagnostic criteria for scurvy were applied here. Rather, we prefer to follow the methodological recommendations by Brickley and Morgan^48^ that apply an inclusive approach to the diagnosis of metabolic diseases. This approach goes beyond numbers and considers the variable nature of bones related to the state of preservation and the degree of disease expression across the skeleton. This allows us to account for intra- and interindividual variability in disease expression. A quantitative approach would be more appropriate for capturing differential expressions of scorbutic lesions across age groups when the osteological sample under study is homogenously well-preserved and has an un-biased age distribution. Such conditions are, however, infrequently satisfied, and the approach advocated for here can facilitate the diagnosis of scurvy with incomplete skeletal remains^48^, as well as with commingled remains. Comparability between paleopathological studies that follow the quantitative approach proposed by Snoddy et al.^40^ and the one applied in the present study can be achieved in all cases where the diagnosis of possible or probable scurvy includes the diagnostic features as listed in Table 2.

A definite diagnosis of scurvy in this study was made when skeletons with any level of completeness displayed macroscopic diagnostic lesions in the form of bilateral and symmetrical abnormal cortical porosities < 1 mm and/or SPNBF on regions of the sphenoid bone, temporal bones, zygomatic bones, maxilla, and mandible which are associated with the muscles used for mastication (i.e., temporalis muscle, masseter, lateral and medial pterygoid muscles, genioglossus and geniohyoid muscles). Macroscopic diagnostic features of the post-cranial skeleton that were considered consist of bilateral and symmetrical SPNBF of the scapulae and ilia.

Radiographic findings of the long bones were taken into consideration if there were macroscopic diagnostic cranial and post-cranial alterations already present. This methodological choice is based on the premise that radiological analysis is not commonly used to diagnose scurvy in clinical cases as they rely on a blood serum test^22^. Moreover, expressions of scurvy may vary across individuals and not all individuals may develop radiographic signs. An absence of radiographic signs therefore does not equate to an absence of disease^22^. Post-mortem damage of long bone metaphyses and epiphyses is also commonly encountered with osteoarchaeological material and can prevent adequate radiological evaluation^48^.

The gross macroscopic examination was aided with a Leica WILD M400 photomacroscope, Heerbrugg, Switzerland; 6.3–32.0 × magnification) which was used to examine details of bone abnormalities. The radiological analysis was achieved using a FCR Velocity by Fujyfilm direct DR equipment for conventional x-rays, with the following parameters: 10–12 mAs with 54–60 keV, DFF 110 cm.

## Data Availability

The datasets used and/or analysed during the current study is available from the corresponding author on reasonable request.
